# Diagnostic Criteria for Fibromyalgia: Critical Review and Future Perspectives

**DOI:** 10.3390/jcm9041219

**Published:** 2020-04-23

**Authors:** Carmen M. Galvez-Sánchez, Gustavo A. Reyes del Paso

**Affiliations:** Department of Psychology, University of Jaén, 23071 Jaén, Spain; greyes@ujaen.es

**Keywords:** fibromyalgia, diagnostic criteria, widespread pain, symptoms severity

## Abstract

Fibromyalgia syndrome (FMS) is a chronic illness characterized by widespread pain and other clinical and emotional symptoms. The lack of objective markers of the illness has been a persistent problem in FMS research, clinical management, and social recognition of the disease. A critical historical revision of diagnostic criteria for FMS, especially those formulated by the American College of Rheumatology (ACR), was performed. This narrative review has been structured as follows: Introduction; historical background of FMS, including studies proposing and revising the diagnostic criteria; the process of development of the ACR FMS diagnostic criteria (1990 and 2010 versions); revisions of the 2010 ACR FMS diagnostic criteria; the development of scales based on the 2010 and 2011 criteria, which could help with diagnosis and evaluation of the clinical severity of the disease, such as the Polysymptomatic Distress Scale and the FMS Survey Questionnaire; relationships of prevalence and sex ratio with the different diagnostic criteria; validity and diagnostic accuracy of the ACR FMS criteria; the issues of differential diagnosis and comorbidity; the strength and main limitations of the ACR FMS criteria; new perspectives regarding FMS diagnosis; and the impact of the novel findings in the diagnosis of FMS. It is concluded that despite the official 2010 FMS diagnostic criteria and the diagnostic proposal of 2011 and 2016, complaints from health professionals and patients continue.

## 1. Introduction

Fibromyalgia syndrome (FMS) is conceptualized as a chronic disorder characterized by widespread and persistent non-inflammatory musculoskeletal pain. Concomitant symptoms usually include fatigue, insomnia, morning stiffness, depression, anxiety, and cognitive problems (forgetfulness, concentration difficulties, mental slowness, memory and attention problems, etc.) [1,2]. Furthermore, the majority of FMS patients usually show predominantly negative affect, including neuroticism, alexithymia, and catastrophizing [3,4,5] and impaired health-related quality of life [6]. 

**Figure 1:**
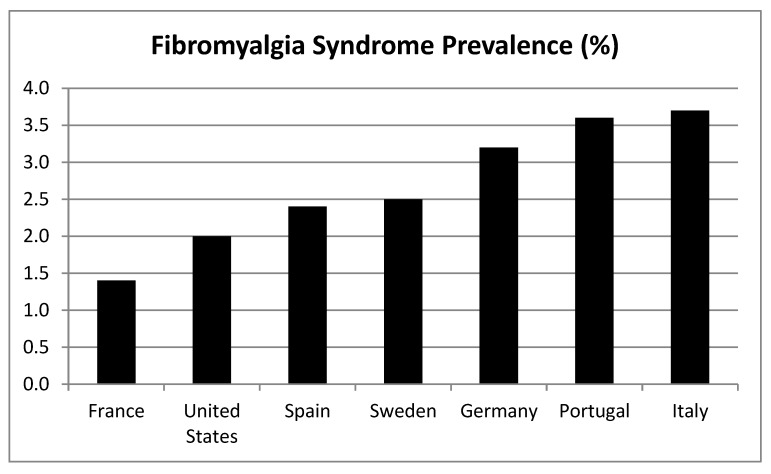
Fibromyalgia syndrome (FMS) Prevalence (%) based on data from the revision by Cabo-Meseguer et al. [7].

The prevalence of FMS is estimated at 2%–4% in the general population, being more frequent in women than in men (see later) [2]. For example, the prevalence in Spain is around 2.4% in the general population [7] (see Figure 1 for prevalence in different countries). As reported by the 2016 EPISER study (prevalence of rheumatic diseases in the adult population in Spain), carried out by the Spanish Society of Rheumatology and published in 2019, the prevalence of FMS in Spain is around 2.45% [8]. We consider that prevalence varies in different countries because the ways in which it is measured are different, the age groups included are also different, and there are also differences in sociocultural beliefs and norms.

Although some factors are known to predispose individuals to FMS (i.e., genes, negative life events, and physical trauma) the etiology of FMS remains unknown [9,10]. One of the most well-supported hypotheses regarding its pathophysiology is the presence of central sensitization to pain and deficits in endogenous pain inhibitory mechanisms [11,12,13]. Evidence supporting this hypothesis includes low thresholds and tolerance of pain, the hyperalgesia and allodynia that characterize FMS, higher responses to dynamic pain protocols that measure pain sensitization, and greater responses in areas of the neuromatrix that process pain during pain evocation in comparison to healthy individuals [14,15,16,17,18]. The existence of small-fiber [19,20] and large-fiber [21] peripheral neuropathy is also supported to some degree.

The lack of objective markers or reliable and valid clinical measures for FMS diagnosis has been a major problem in FMS research and clinical management [22,23,24]. Until the etiology or pathophysiology is better understood, the diagnosis should rely upon clinical assessment and patient reports. Thus, the subjective nature of FMS symptoms and lack of objective markers have undermined disease comprehension, healthcare, and social acceptance. 

Usually, the diagnosis of FMS may take years to be completed, with patients visiting several medical specialists in that time.

In this context, the development of objective and reliable diagnostic criteria is of primary importance for improving FMS research and understanding of the disease. This review of FMS criteria is focused on adults with FMS, not the pediatric population.

## 2. Historical Background on the Study of FMS

It is believed that the disease now called FMS first began to be recognized and studied in the 16th century. The first description of the disease was provided by Guillaume de Baillou in 1642, who used the term “muscular rheumatism” [25]. The term “fibrositis” was coined by W.R. Gowers in 1904 [26] to describe pain on touching by the fingertips of muscles hardened by inflammation of the fibrous tissue. This author also noted the existence of spontaneous pain and hypersensitivity to mechanical pressure, as well as fatigue, sleep disorders, and aggravation of muscular symptoms by cold weather and overexertion. Also in 1904, R. Stockman established the pathological basics for the fibrous tissue inflammation theory of W.R. Gowers. This author noted the existence of painful nodules, where hyperplasia was observed in inflammatory connective tissue [27]. This theory ended up being refuted because biopsies performed on muscle tissues did not provide evidence of inflammation. Therefore, some authors believe that these initial uses of the term fibrositis cannot be considered synonymous with FMS [28]. E.W. Boland (1947), in the absence of physical findings corresponding to the symptomatology, modified the concept and proposed the term “psychogenic rheumatism”, defined as musculoskeletal expression of functional disorders, stress states, or psychoneurosis [29].

Subsequently, W. Graham (1953) used the term “fibrositis” to denote a pain syndrome in the absence of any specific organic disease [30]. The term fibrositis, when ascribed this meaning, could be considered as synonymous with FMS as we understand it today. E.F. Traut (1968) conceptualized “muscular fibrositis” or “non-articular rheumatism” as a syndrome consisting of generalized pain, tiredness, sleep disorders, and palpation pain in trigger areas, which included the soft tissues of the neck, shoulder, elbow, carpal tunnel, palms (Dupuytren’s contracture), and lower back area [31]. 

In 1976, P.K. Hench coined the term fibromyalgia (from the Latin word “fibro”, which means fibrous tissue, and the Greek words “mio” (muscle) and “algia” (pain)) as a form of non-articular rheumatism [32]. The FMS concept was widely criticized [33], being considered a circular concept [34,35], a medicalized disorder [35,36], or a socially constructed concept [37,38]. These first criticisms mark the origin of the debate about the nature and legitimacy of FMS as a reliable and valid medical disorder.

In 1977, H.A. Smythe and H. Moldofsky continued the work of P.K. Hench and proposed the first measure for evaluating FMS in their work entitled “Two Contributions to the Understanding of the Fibrositis Syndrome” [39]. They described the illness, and proposed diagnostic criteria, based on what they considered as its key features: non-refreshing sleep and tender points to pain. However, these 1977 criteria had several limitations. Despite unrefreshed sleep, fatigue, and widespread pain being required for the diagnosis, no definition or assessment methods were recommended for these conditions. On the contrary, the tender point count criterion was explicitly defined, i.e., a requirement that 12 of 14 anatomic sites be positive for tenderness. This explains why the tender point count (based on digital palpation) was finally considered the most relevant feature of FMS, while the other proposed symptoms were ignored. Later, other authors set different numbers of tender points (e.g., [40,41]; see Table 1). 

However, it was not until 1981 that the medical community started accepting the disease under the term “fibrositis” or “fibromyalgia” [42]. In fact, in 1981, Yunus et al. introduced the following formal set of criteria to diagnose primary FMS: (1) Obligatory Criteria: (A) presence of aching, pain, or stiffness in three anatomical areas for at least 3 months, (B) absence of causes to explain the condition, e.g., traumatic (due to repetitive or more direct trauma), rheumatic (including degenerative), infective, endocrine, or malignant, with normal laboratory tests. (2) Major Criteria: presence of at least five typical and consistent tender points. (3) Minor Criteria: (A) modulation of symptoms by physical activity (aggravated due to physical inactivity and relieved with moderate physical activity), (B) modulation of symptoms by weather (i.e., worsening of symptoms due to cold, humid weather, and relief of them by heat) or time factors (i.e., worsening of symptoms in the morning and the evening), (C) aggravation of symptoms by anxiety or stress, (D) poor sleep, (E) general fatigue or tiredness, (F) anxiety, (G) chronic headache, (H) irritable bowel syndrome, (I) subjective swelling, and (J) numbness (symptoms can be also relieved by reduction of stress and vacations). All primary FMS patients must satisfy the two obligatory criteria, as well as the major criterion plus at least three minor criteria. If the patient has only 3 or 4 tender points, then five minor criteria must be met [42]. For cases wherein a secondary condition (e.g., inflammation) produces the syndrome, the term secondary fibrositis was proposed. The criteria of Yunus et al. [42] were the first in which symptoms began to play a more central role in diagnosis, thus furthering the understanding and treatment of FMS. The primary versus secondary distinction disappeared some years later. Yunus et al. [42] also suggested that primary FMS should be considered according to the presence of its own characteristic features and not diagnosed just by the absence of other recognizable conditions; this continues to be important in current primary attention care [49] and can be useful for early risk assessments for FMS. During the 1980s, many other formal and ad-hoc criteria sets were introduced [50].

In 1987, the American Medical Association accepted FMS as a disease. As a result of this recognition, the American College of Rheumatology (ACR) created a committee to establish the diagnostic criteria for FMS [1].

## 3. Development of the ACR FMS Diagnostic Criteria

**Figure 2:**
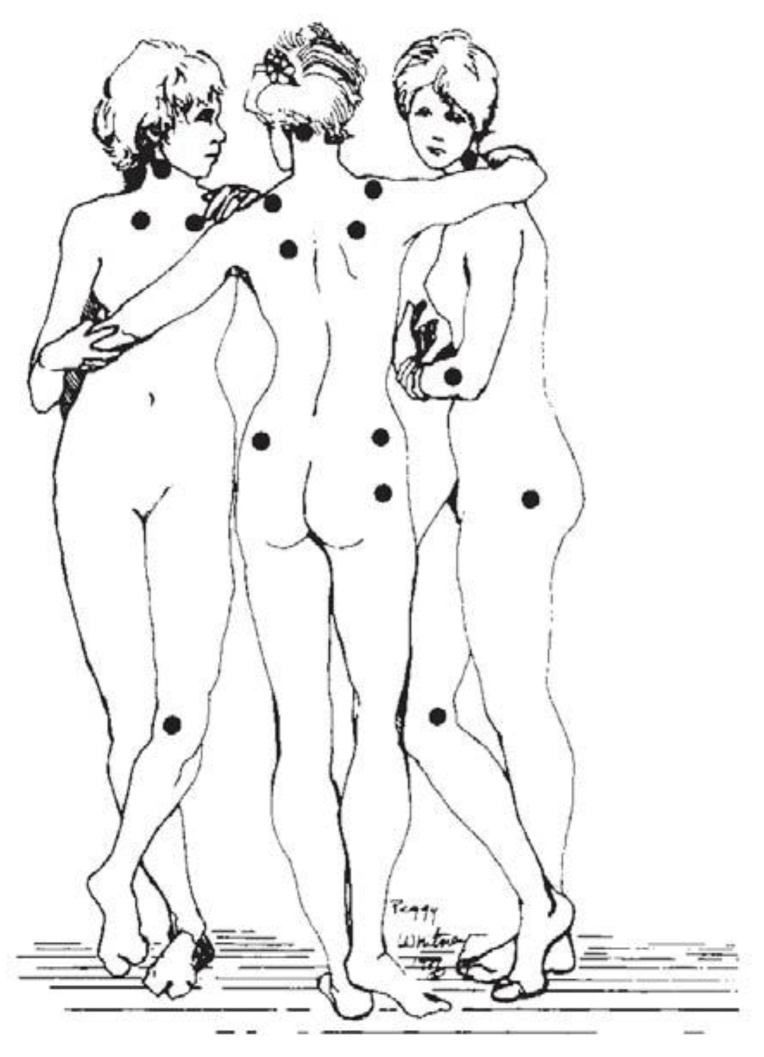
Location of the 18 tender points established as criteria for FMS diagnosis by the American College of Rheumatology (ACR) [1]. Image based on the original “The Three Graces” by the French painter Jean-Baptiste Regnault (1793).

In 1990, the ACR proposed the first official diagnostic criteria and decided to use the term FMS instead of fibrositis [1]. Pain in response to a pressure up to 4 kg/cm^2^ (exerted with an algometer) was evaluated in 18 body bilateral points; to make the diagnosis, it was necessary to elicit a painful response in 11 of these points [1] (see Figure 2). Moreover, six control sites (three pairs) were included in the physical examination: the forearm at the distal dorsal third of the forearm, the thumbnail, and the midfoot, i.e., at the midpoint of the dorsal third metatarsal. Furthermore, a history of generalized pain for at least 3 months was required in some region of the axial skeleton and in at least three of the four body quadrants (or, exceptionally, in two opposing quadrants with respect to the two axes of body division). No distinction between primary versus secondary FMS was made. The 1990 criteria have been criticized because of their limited predictive validity with regard to clinical pain the difficulties of applying and standardize pressure algometry in primary health care, which makes the tender point count impractical in clinical settings; the lack of consideration of important symptoms such as sleep difficulties and fatigue; and the conceptualization of FMS as an “all or nothing” disorder, rather than being on a continuum [2,50,51,52]. In addition, the 1990 ACR criteria did not deal adequately with patients who once met the 1990 criteria but due to improvements or measurement error, no longer satisfied the diagnostic criteria; this applied to about 30% of patients previously diagnosed with FMS [2]. Thus, these criteria failed to meet the requirements of physicians, researchers, and patients.

In spite of their limitations, the 1990 ACR criteria brought official recognition to FMS. In 1992, FMS was included in the International Classification of Diseases (ICD), received international recognition as a source of disability and funding from research and government institutions, and gained academic recognition. In 1992, the World Health Organization (WHO) also recognized FMS as a disease, and it was classified as a non-joint type of rheumatism under the code M.79.7 of the ICD. Therefore, the 1990 ACR diagnostic criteria helped legitimize the syndrome [50]. 

**Figure 3:**
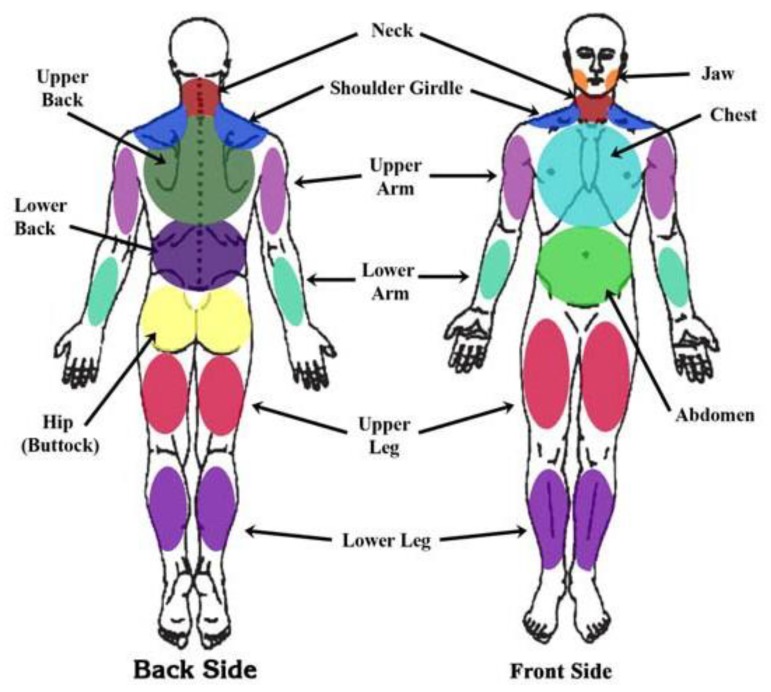
Body areas included in the Widespread Pain Index (WPI) scale of 2010 FMS ACR diagnostic criteria [2].

In 2010, the ACR proposed a new version of the diagnostic criteria based exclusively on the use of two scales: the Widespread Pain Index (WPI) and the Symptom Severity (SS) Scale. The WPI comprises a list of 19 painful areas (score range: 0–19; see Figure 3). Patients report whether each point hurts her/him. The SS includes two parts: Part SS2a (with a 4-point Likert scale; 0 to 3) evaluates the severity of fatigue, waking unrefreshed, and cognitive symptoms. Part SS2b consists of a checklist of 41 symptoms (irritable bowel syndrome, fatigue/tiredness, muscle weakness, Raynaud’s, ringing in ears, etc.). Patients have to state whether or not they have these symptoms. Based on the number of symptoms, patients are included in one of four score ranges: 0 symptoms (score of 0), 1 to 10 symptoms (score of 1), 11 to 24 symptoms (score of 2), and 25 or more symptoms (score of 3). The SS (score range: 0–12) is derived from the sum of the results of parts SS2a (score range: 0 to 9) and SS2b (score range: 0–3). To diagnose FMS, one of these two conditions must be fulfilled: a WPI ≥ 7 and SS ≥ 5, or a WPI between 3 and 6 and SS ≥ 9. Similar to the 1990 criteria, it is mandatory that symptoms be present for at least 3 months [2]. The 2010 criteria always require a physician’s assessment and should never be replaced by patient self-report. The 2010 ACR criteria have been validated in Iranian [53], Japanese [54], French [55], Turkish [56], and Spanish [57] populations, but have been criticized due to the requirement for a physician to evaluate symptoms, which limits usefulness in large-scale studies [58] and leads to possible bias due to the subjectivity of physician assessments [50]. The 2010 ACR criteria have also been criticized due to the absence of a tender point count [59,60]. In reply, Wolfe et al. [2] showed that SS scores correlate better with WPI scores (0.73) than with tender point count (0.68). 

## 4. Revisions of the 2010 ACR FMS Diagnostic Criteria

In this section, two other proposals of diagnostic criteria (2011 and 2016 proposals) are discussed. However, only the 1990 and the 2010 criteria have been officially recognized by ACR. In 2011, Wolfe et al. [61] revised and modified the 2010 ACR diagnostic criteria (also called the 2011 criteria in Wolfe et al., 2016, and in the current revision). These modifications were made to facilitate its use in epidemiological or community studies but not for self-diagnosis in the clinical context [62]. According to Wolfe et al. [61], the requirement for a physician examination was a major barrier to understanding FMS prevalence and characteristics based on large epidemiological or community studies. As the majority of the WPI and SS ACR 2010 items can be self-reported, Wolfe et al. [62] modified both scales, but mainly the SS, to allow patient self-administration. The modification consisted of substituting the physician’s estimate of the extent of somatic symptoms with the sum of three specific self-reported symptoms. The SS was modified by replacing the checklist of 41 symptoms with a somatic symptoms score (score range: 0–3) representing the sum score for three items: the presence or absence of (1) headaches, (2) pain or cramps in the lower abdomen, or (3) depression symptoms (including depressive symptoms, feelings of depression, or depressed mood). Depression in this context does not indicate a psychiatric diagnosis of depression. The modification of SS was done because it did not seem reasonable to ask patients to evaluate their own somatic symptoms. As in the 2010 version, patients were also asked to report painful areas using the WPI. The change in somatic questions and method of administration represents the essential differences between the 2010 and 2011 ACR criteria. One of the main limitations of the 2011 criteria was the possibility that patients might have another primary disorder that could be causing their pain [62].

Segura-Jiménez et al. [63] validated the 2011 criteria in Spanish FMS samples. A sensitivity of 88.3% and specificity of 91.8% for discriminating between FMS patients and healthy controls were reported [63]. The 2011 criteria have been also validated in Japanese, showing a sensitivity of 64% and specificity of 96% for discriminating between FMS patients and a group of rheumatoid arthritis and osteoarthritis patients [64].

In 2016, a systematic review of the 2010 and 2011 ACR criteria was performed [58]. Some limitations were reported, such as imprecise language and definition, a lack of clarity regarding FMS diagnosis when it is accompanied by other diseases, different assessments between the 2010 and 2011 criteria, inability to exclude some regional pain syndromes, and limitations in the validity and reliability of the diagnosis in a clinical context (but not in the research context). However, a strong point was the high levels of agreement in sensitivity and specificity between the 1990, 2010, and 2011 ACR criteria [58]. 

To diagnose FMS using the proposed 2016 criteria, the following conditions must be fulfilled. (1) Presence of generalized pain, defined as pain in at least four of five regions (four quadrants and axial). According to this definition, jaw, chest, abdominal, headache, and facial pains should not be included in the quadrant or regional definition of generalized pain. Wolfe et al. [58] showed that the use of this modified widespread pain criterion (termed “generalized pain” to distinguish it from the 1990 widespread pain criterion that included pain above and below the diaphragm, and right and left and central and peripheral pain) requiring pain in four of five pain regions eliminated misclassification of regional pain syndromes as FMS. In fact, the widespread anterior pain criterion showed a misclassification rate of 1.8%, while the proposed 2016 generalized pain criterion (pain in 4–5 regions) showed a misclassification rate of 0.4%. Misclassification seems to occur because the WPI does not consider the spatial distribution of the painful sites; a requirement of satisfying a widespread pain criterion could solve this problem. (2) Symptoms have been present at a similar level for at least 3 months. (3) WPI ≥ 7 and SS ≥ 5 or WPI of 4-6 and SS ≥ 9. (4) A diagnosis of FMS is valid irrespective of other diagnoses, and a diagnosis of FMS does not exclude the presence of other clinically important illnesses. 

**Figure 4:**
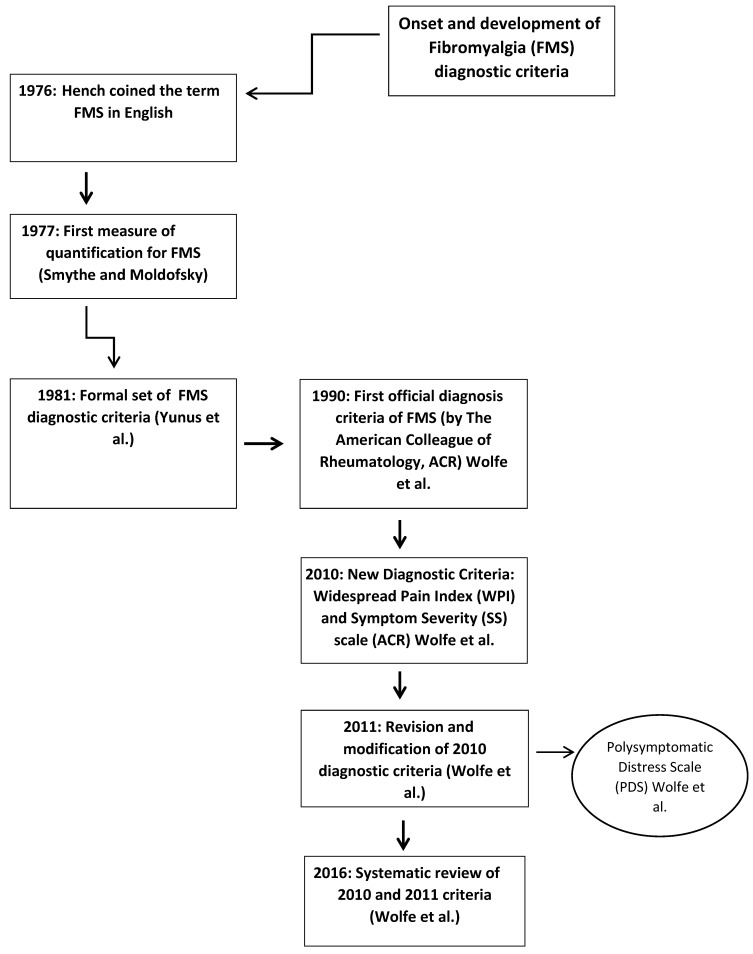
History of the development of FMS Diagnostic Criteria.

The 2016 proposal criteria propose combining the 2010 ACR criteria and 2011 proposal into a single set that can be used by physicians or patients. It has been suggested that the 1990 criteria and 2016 proposal measure FMS in somewhat different ways. The 1990 criteria emphasize peripheral allodynia (tender points), while the 2016 criteria focus on central pain perception and distress [65]. The 2016 criteria were designed to replace the 2010 ACR criteria and 2011 proposal (see Figure 4 for a review of the crucial considerations in the conception and development of FMS diagnostic criteria). 

## 5. The Polysymptomatic Distress Scale and the FMS Survey Questionnaire 

The 2010 ACR diagnostic criteria and the diagnostic proposal criteria of 2011 and 2016 eliminated the previous 1990 tender point exam and defined FMS as a multi-symptom disorder. In this context, and based on the 2010 ACR criteria, the Polysymptomatic Distress Scale (PDS, score range: 0–31) was developed. The PDS is computed as the sum of the WPI (score range: 0-19) and SS (score range: 0–12) scales [50]; the PDS has also been called the Fibromyalgianess Scale (FS) [50]. However, some authors propose that it would be preferable to use the term “central sensitivity score” rather than “polysymptomatic distress scale”, “fibromyalgianess scale”, or “FMS survey score” [66]. Based on the PDS, we can classify FMS patients into different severity categories: none (0–3), mild (4–7), moderate (8–11), severe (12–19), or very severe (20–31) [67]. Wolfe et al. [67] recognized that experts may consider a continuous scale important, but for ordinary use, the authors decided to use severity categories. 

A positive FMS diagnosis will always have a PDS score of at least 12, but not all patients with a score ≥ 12 will satisfy the FMS criteria because there is a small degree of misclassification (sensitivity 95%, specificity 93%) [67]; for that reason, Wolfe et al. [50] reported that a PDS score ≥ 13 was the optimum cut-off. However, the optimum PDS cut-off point depends on the proportion of people who satisfy the 2010 ACR criteria or 2011 proposal, and on the distribution of PDS scores among study participants [67]. Therefore, it was suggested that the previous diagnostic criteria (i.e., the 2010 or 2011 criteria) be used, rather than the PDS categories, for individual patient diagnosis, and that the PDS be used to evaluate severity and treatment effects [67]. The PDS seems to be a useful questionnaire to support FMS diagnosis because it is easy to apply, provides a measure of severity, and can be used in various types of patients, not just those with FMS, given that it is a continuous measure [67]. 

As the majority of the WPI and SS items are self-reported, Wolfe et al. [50] also proposed the FMS Survey Questionnaire (FSQ) to facilitate epidemiological studies. The FSQ concerns the following: (1) severity of fatigue, cognitive problems (specifically attention, concentration, and memory problems), and non-restorative sleep during the last week (score range: 0–3), (2) three yes/no questions about problems caused by the presence of pain or cramps in the lower abdomen, headache, or depression symptoms during the last 6 months, (3) the presence or absence of pain in 19 painful areas, and (4) a yes/no question concerning whether 1, 2, and 3 have all been present for at least 3 months. The FSQ has been validated in Spanish [68], German [69], Japanese [64], and Persian [70] populations. Carrillo-de-la-Peña et al. [68] found that the FSQ showed good internal consistency (0.68 for the SS and 0.85 for the PDS) and a high degree of convergence with clinician diagnoses of FMS, following either the 1990 (concordance rate of 83.02%) or the 2010 (concordance rate of 100%) ACR criteria [71]. However, it is recommended that the FSQ not be used for self-diagnosis, or as a surrogate for physician diagnosis [69].

## 6. Associations of Prevalence and Sex Ratio with the Different Diagnostic Criteria

The prevalence of FMS appears to differ according to the diagnostic criteria used. The 1990 criteria have been considered as stricter than the 2010 criteria, such that only more severely affected patients are identified [2]. Studies recruiting FMS patients according to the 1990 ACR criteria reported higher mean WPI and SS scores than studies in which patients were recruited using the 2010 ACR criteria [2,72]. Nevertheless, a study which divided FMS patients into adapted (i.e., lower levels of psychological distress, catastrophizing, and fatigue) and maladapted profile (including problems in resilience, catastrophizing, and memory) found in the adapted profile subgroup a greater percentage of patients fulfilling the 1990 ACR criteria but not the 2011 criteria, whereas the opposite occurs in the maladapted profile subgroup. The authors explained that the change in focus of the criteria from pain towards a wider view on multiple FMS symptoms might lead to an inclusion of more patients with a maladapted profile in the 2011 criteria [73]. In accordance with this explanation, patients with a maladapted profile (that includes features other than pain, like depression, stress, catastrophizing, etc.) can fulfill more easily a multidimensional concept of FMS than one more focused on specific body pain areas. The different ways of administration of the criteria may also be a relevant issue. While the 1990 criteria are applied by a physician, the 2011 criteria are self-administered. Self-reported measures may involve a negative affectivity component (i.e., anxiety, depression, and catastrophizing) that could increase the intensity and frequency of self-reported symptoms regardless its actual levels [74,75], which could explain the inclusion of more patients with a maladapted profile with the use of 2011 criteria.

Furthermore, it appears that tender point count is biased toward women, who are more sensitive to tender point pressure than men. Additionally, tender point exam can be influenced by subjective distress, which is more frequent in women than men [76]. Prevalence appears to be higher when the 2010 criteria are applied, especially in comparison to clinician-based FMS diagnoses [77]. Using the self-administered 2011 criteria, the prevalence is also higher, reaching 5.4% [77]. 

Traditionally, FMS has been considered a female-predominant disorder. However, the sex ratio varies significantly as a function of the criteria applied. A greater proportion of men are diagnosed with FMS when the 2010 ACR criteria are used. In fact, with the 1990 ACR criteria, a female-to-male ratio of 13.7:1 was observed, while the ratio with the 2010 ACR criteria was 4.8:1, and even lower with the 2011 proposal (2.3:1) [77]. Recent studies [78] suggest that the perception of FMS as almost exclusively a women’s disorder (the traditionally accepted female ratio was ≥ 90%) is not supported by data from unbiased studies. With the use of validated criteria and unbiased selection of patients, the female proportion of cases was ≤ 60% [78]. These results suggest the existence of significant bias in patient selection and diagnosis, both in the clinical context and in scientific publications. Bias leads professionals to underestimate FMS prevalence in men and overestimate it in women [79]. Therefore, Wolfe et al. [78] recommend the use of the 2016 FMS criteria for clinical diagnosis and epidemiological studies due to its updated scoring and requirement for generalized pain.

## 7. Validity of the ACR FMS Criteria

With the 1990 ACR criteria, FMS can be distinguished from the other rheumatic conditions with a sensitivity of 88.4% and specificity of 81.1% [1]. With the 2010 ACR criteria, a sensitivity of 96.6% and a specificity of 91.8% for discriminating FMS from rheumatic arthritis (RA) and osteoarthritis (OA) was reported [2]. In Spanish samples, a sensitivity of 85.6% and specificity of 73.2% were reported for discriminating FMS from RA and OA [57]. For FMS patients recruited using the 1990 ACR criteria, 100% sensitivity and 81% specificity were found in the discrimination between FMS and RA, again in Spanish samples using the 2010 ACR criteria [72]. Furthermore, all patients recruited with the 1990 criteria fulfilled the 2010 criteria, suggesting good diagnostic agreement between both sets of criteria [72]. When the cut-off values of the 2010 ACR criteria were increased up to WPI ≥ 14 and SS ≥ 7, a specificity and overall diagnostic accuracy of 100% were achieved [72]. Studies using logistic regression analysis showed that the ability to discriminate between FMS and RA patients was greater for the WPI (95.9% overall accuracy) than for the SS (87.1% overall accuracy) [72]. 

## 8. Differential Diagnosis and Comorbidity

The need to exclude other related diseases and perform a differential diagnosis has been a somewhat contentious issue with respect to the 2010 ACR criteria [80]. The 2010 criteria stated, “We would like to point out that implicit in the 1990 ACR classification criteria was the requirement that clinical examination and clinical judgment had excluded other causes of chronic widespread pain, and such an exclusion is also implicit in the proposed diagnostic criteria. It is important for physicians to perform an appropriate clinical assessment to exclude other diagnoses and/or to identify potential coexisting rheumatic diseases that may require treatment themselves” (Wolfe et al. [2], page 609). However, Wolfe et al. [72] indicate that exclusionary tests are not needed for FMS diagnosis and that FMS remains a valid construct irrespective of other comorbid diagnoses. The strength of the 2016 criteria is the elimination of the previous, confusing recommendations related to diagnostic exclusion. In the 2016 criteria, a diagnosis of FMS does not exclude the presence of other clinically important illnesses [58]. This approach to FMS diagnosis allows us to diagnose patients with FMS who previously would not have fulfilled them due to their comorbidities. It is important to note that the absence of exclusion criteria does not mean that post hoc exclusions cannot be added for research studies (e.g., surveys and epidemiological studies) or clinical trials (e.g., pharmaceutics studies). Therefore, the FMS exclusion criteria do not differ from those for the other rheumatic diseases [58]. However, in spite of the previous recommendation [2,49,50,58,62], the diagnosis of FMS continues to be fundamentally based on the exclusion of other similar diseases in the clinical context. 

Nevertheless, there are certain situations where exclusion would be useful (e.g., in patients with multiple bone metastases and anemia, extreme hyperthyroidism, regional pain syndrome, and generalized fatigue.) [72]. It is also important to consider that rheumatic diseases do not usually cause pain that could be confused with FMS, in spite of frequently coexisting with FMS [72]. Moreover, we should pay attention to the presence of other chronic overlapping pain conditions and mental disorders in FMS and take them into consideration for improving its treatment and management [81]. 

## 9. Limitations of the ACR FMS Criteria

Both the 2010 ACR criteria and 2011 proposal show a change in the conceptualization of FMS— or at least in its classification—from being fundamentally a pain syndrome to a multi-symptom syndrome [77]; this is relevant to understanding and treating the disease.

However, in spite of the current ACR criteria, a considerable rate of misdiagnosis of FMS in the general population has been reported [81,82,83]. Patients are at risk of being overdiagnosed or underdiagnosed [81], which is a serious public health problem that can lead to overtreatment [84,85], or inadequate treatment of FMS patients not recognized as such [86]. In spite of the 1990 ACR criteria being replaced by the 2010 criteria and later diagnostic proposals being made (i.e., the 2011 and 2016 criteria), in clinical settings, the majority of health professionals continue to employ digital palpation, in which controlling the level of pressure exerted is difficult, and do not systematically apply any of the criteria proposed by the ACR [58,79,86,87]. Clinician-based FMS diagnosis may lead to bias due to the subjective nature of the symptoms assessed [50]. This shows that there is no clear consensus regarding the concept and diagnosis of FMS among medical professionals. 

Another limitation of current FMS criteria (including the official 2010 criteria and the proposals of 2011 and 2016) is a lack of sufficient recognition of psychological, environmental, and sociocultural factors, in spite of the fact that they play an important role in the onset, maintenance, diagnosis, and treatment of FMS [82,83]. It is well-established that neurobiological mechanisms play an important role in FMS pain; however, the diagnosis of FMS is also related to social and cultural factors, and its diagnosis can influence social status, the availability of medical care, and access to private and government disability payments [86]. Therefore, the diagnosis of FMS seems to involve complex processes, such that a biopsychosocial approach is required. 

Currently, despite advances in our understanding of FMS, many patients report feeling misunderstood by relatives, friends, health professionals, and society in general; they live with a stigmatized and invisible disorder [88], which points to the need for continued research to for complete legitimization and acceptance of FMS. Some authors proposed the term invalidation to refer to this constellation of features that includes no acceptance by others, misunderstanding, disbelief, rejection, denying, stigmatization, and suspicion that the problem is exaggerated or due to psychological reasons [89,90]. Additionally, these authors proposed the Illness Invalidation Inventory to examine antecedents and consequences of invalidation as well as the effect of treatments targeting invalidation [90].

## 10. New Perspectives on FMS Diagnosis

Taking the previous ACR diagnostic criteria limitations into account, Arnold et al. (2019) [91,92] have proposed an alternative for the FMS diagnosis. This alternative is included in the ACTTION-APS Pain Taxonomy (AAPT) developed by The Analgesic, Anesthetic, and Addiction Clinical Trial Translations Innovations Opportunities and Networks (ACTTION), a public–private partnership with the U.S. Food and Drug Administration (FDA) and the American Pain Society (APS), in order to develop a clinically useful and consistent diagnostic system for chronic pain disorders, including FMS. In the case of FMS, the AAPT established an international working group of clinicians and researchers with expertise in FMS, to develop new diagnostic criteria. 

This new diagnostic proposal is based on the conceptualization of FMS as a dimensional syndrome which includes five dimensions: (1) Core Diagnostic Criteria, defined as the presence of pain in six or more body sites from a total of nine possible localizations, sleep disturbance, and fatigue; (2) Common Features, like tenderness, dyscognition (e.g., trouble concentrating, forgetfulness, and disorganized or slow thinking), musculoskeletal stiffness, and environmental sensitivity or hypervigilance; (3) Common Medical and Psychiatric Comorbidities like chronic fatigue syndrome, irritable bowel syndrome, chronic pelvic pain, interstitial cystitis, orofacial conditions, chronic headaches, depression, anxiety disorders, central sleep apnea, restless leg syndrome, etc.; (4) Neurobiological, Psychosocial and Functional Consequences, which includes general outcome, functional disability, social and medical cost of FMS, morbidity, and mortality; and (5) Putative Neurobiological and Psychosocial Mechanisms, Risk Factors, and Protective Factors that focus on risk factors, comorbidities, and pathophysiology aspects. The strength of this proposal is the inclusion of risk factors, course, prognosis, and pathophysiology into FMS criteria. These new criteria have not yet been validated, and no data are still available about their diagnostic accuracy. However, it has been suggested that with the use of these criteria prevalence of FMS would increase significantly as well as the proportion of false positives [93]. Thus, more studies are required to assess the feasibility, reliability, and validity of these new proposed FMS diagnostic criteria [91].

## 11. Impact of the Novel Findings in the Diagnosis of FMS 

The aforementioned findings in FMS diagnosis reinforce the need to advance in this field to benefit not only FMS patients and relatives but also reduce the cost of FMS for the health system. The existence of some biases, including gender ones, in FMS diagnosis [50,79] arise as one of the most relevant findings given that it might be the origin of the social representation of FMS as a female illness. The female connotation of FMS has a negative impact on male FMS patients, who could have problems accepting their medical condition due to the gender stereotype about FMS. Furthermore, this gender bias also may be leading to less attention paid to the male population with FMS.

Moreover, it is necessary to revise the ACR 2010 criteria and take decisions about the 2016 FMS diagnostic proposal, in order to establish more adequate diagnostic criteria. Ten years after the endorsement of the last official FMS diagnostic criteria, it may be a good moment to perform a revision by ACR. It is also crucial to insist on the need that physicians and health workers receive training to perform FMS diagnosis, given that in many cases, the physician does not use any official criteria and base their diagnosis on their clinical judgment.

The possibility of incorporating some objective or reliable measures associated with the pathophysiology of FMS, combining physician test with questionnaire evaluation, might help to increase diagnosis reliability and validity. The more consensual hypothesis about pathophysiology of FMS is the existence of central sensitization to pain [12,13]. There is a protocol to easily measure central sensitization with the assistance of an algometer. This protocol is based on the increase in pain perception due to slowly repeated evoked pain (SREP) [14]. Unlike other sensitization protocols such as Temporal Summation of Pain, SREP sensitization is reliably observed in FMS patients but not in healthy individuals or patients with other chronic pain conditions of peripheral origin like rheumatoid arthritis (RA) [15]. SREP sensitization allows for a global diagnostic accuracy of 85.4% in discriminating FMS patients from healthy individuals and 87.7% in discriminating FMS from RA patients. The combination of such a central sensitization index together with core FMS symptoms may enhance diagnostic accuracy. In a recent study, the combination of SREP sensitization jointly with fatigue and insomnia allows for a global diagnostic accuracy of 99% (98% of sensitivity and 100% of specificity) [94]. The incorporation of diagnosis rules of objective or reliable markers that are easy to incorporate in common health services, like SREP, may improve FMS detection. Future studies should follow this line of research.

## 12. Conclusions

Despite the new official 2010 FMS diagnostic criteria and the diagnostic proposals of 2011 and 2016, complaints from health professionals and patients about the way the disease is diagnosed continue, and a significant proportion of patients who do not fulfill the criteria are nevertheless severely affected. Furthermore, in many cases the FMS diagnosis is fundamentally based on the exclusion of other similar diseases; in spite of that practice not being recommended because of its lack of precision and the high possibility of misdiagnosis. Moreover, current diagnostic criteria do not take sufficient account of psychological, environmental, and sociocultural factors, despite the fact that they play an important role in the onset, maintenance, diagnosis, and treatment of FMS. There is also a considerably high rate of misdiagnosis among the general population. In spite of the 1990 ACR criteria being replaced by the 2010 criteria and later diagnostic proposals being made (i.e., the 2011 and 2016 criteria), in clinical settings, the majority of health professionals continue to apply digital palpation, wherein it is difficult to control the level of pressure exerted, rather than systematically applying any of the criteria proposed by the ACR. This situation, together with clinician bias regarding patient sex and prevalence, seems to be responsible for misdiagnosis. This is a serious public health problem that can lead to overdiagnosis and overtreatment, or inadequate treatment of FMS patients who are not recognized as such. In addition, some clinicians have a tendency to diagnose the illness more often in women than in men. It is essential that genuine acceptance of FMS by health professionals and general society be achieved.

## Figures and Tables

**Table 1:** Different approaches to Tender Points in FMS Diagnosis.

Authors	Required Tender Points
Smythe & Moldofsky [39]	12 of 14
Bennett et al. [40]	10 of 25
Yunus et al. [42]	3–5 of 40
Payne et al. [43]	4 of 14
Wolfe & Cathey [44]	7 of 14
Campbell et al. [45]	12 of 17
Wolfe et al. [1]	11 of 18
Greenfield et al. [46]	≥7
Raspe et al. [47]	≥17 tender points and ≤2 control tender points
Borenstein [48]	11 of 18

Note: Tender points are referred to as areas of tenderness occurring in different anatomic sites which hurt when they are pressed.
